# Vaccine equity: there is no time to waste

**DOI:** 10.2471/BLT.21.287655

**Published:** 2022-01-01

**Authors:** Akhil Bansal

**Affiliations:** a Stanford University, Existential Risk Initiative, 450 Serra Mall, Stanford, CA, 94305, United States of America.

Inequitable distribution and access to vaccines for the coronavirus disease 2019 (COVID-19) have compounded the pandemic and caused excess deaths as well as additional economic, social and political disruption.^1^ Over 7.8 billion doses of vaccines have been administered as of 27 November 2021, yet less than one fourth of these doses were given in lower-middle-income or low-income countries^2^ although these countries are carrying a disproportionate burden of morbidity and mortality from the COVID-19 pandemic. Studies estimate that the excess mortality from the COVID-19 pandemic is 35 times greater for low-income countries than for high-income countries.^3^

The omicron variant of the severe acute respiratory syndrome coronavirus 2 emerged in the last week of November 2021. Low vaccination coverage, partly due to inequitable distribution, has likely fuelled the emergence of this variant, since it is well established that viruses are much more likely to mutate in an environment where rates of vaccination are low.^4^

The pandemic will not end until global vaccinations are available;^5^ therefore, understanding how to best distribute vaccines equitably in all countries is crucial. Although high-income countries made significant commitments to deliver vaccines through the COVID-19 Vaccine Global Access (COVAX) initiative and others, these commitments have not yet materialized. COVAX is falling short of its forecasts for 2021, with only 537 million vaccines delivered of the 2 billion targeted for 2021.^6^ Furthermore, many countries have prioritized giving booster vaccinations to their populations rather than sending vaccines abroad, although the World Health Organization (WHO) called for a moratorium on booster doses until at least the end of 2021.^7^

Given the unequal delivery of vaccines, countries should have the right to produce their own vaccine supplies. This principle underpins the campaign to temporarily waive intellectual property protection on COVID-19 vaccines. The proposal is known as the “TRIPS waiver” for Trade-Related Aspects of Intellectual Property Rights. The waiver would allow factories around the world to freely collaborate and supply COVID-19 vaccines, as well as other treatments, tests and medical tools. Doing so is essential in facilitating global access to the COVID-19 vaccines and treatment, particularly for those living in the lowest income countries.

India and South Africa proposed the TRIPS waiver in October 2020; it has since been backed by over 100 countries.^8^ The initiative has also received support from international organizations such as WHO, *Médecins Sans Frontières*, Amnesty International and the Joint United Nations Programme on HIV/AIDS. However, the proposal does not have the support of the pharmaceutical industry, nor that of many high-income countries, including Australia, Canada, Japan, Norway, Switzerland, the United Kingdom of Great Britain and Northern Ireland and countries of the European Union.^9^ These countries’ biggest concern is that such waivers provide a shortcut to competitors looking to acquire expensive technology.^10^ However, this argument wears thin given the scale of the pandemic.

Although the need for such a waiver has existed for over a year, the emergence of the omicron variant has strengthened the need for the TRIPS waiver. The United States of America reiterated endorsement of the TRIPS waiver on 26 November 2021. On Monday 29 November, a coalition of nursing unions from 28 countries and territories – representing over 2.5 million health-care workers – filed a complaint to the High Commissioner for Human Rights, accusing the countries that oppose the TRIPS waiver of a rights violation.^11^

The emergence of the omicron variant highlights the imbalance and inequity of access to COVID-19 vaccines and treatments. While countries in southern Africa have been transparent and forthcoming with scientific information regarding the omicron variant, high-income countries have held tightly to their intellectual property for the COVID-19 vaccines and other technologies.

Understandably, such behaviour has caused public outrage.^12^ No More Pandemics, a grassroots non-profit organization that advocates vaccine equity, is encouraging people in the United Kingdom to write to their members of parliament to persuade them to endorse the TRIPS waiver. 

How to best push such a waiver through is unclear. Perhaps the work of international organizations, organized advocacy from respected professional bodies or grassroots campaigning from organizations such as No More Pandemics would be the most effective. Ultimately, we all must campaign for vaccine equity and the TRIPS waiver, because until the entire world has equal access to vaccines, the pandemic will continue to threaten the lives and livelihood of people globally.
